# Comparative Study Between Coaptive Film Versus Suture For Wound Closure After Long Bone Fracture Fixation

**DOI:** 10.5704/MOJ.1303.017

**Published:** 2013-03

**Authors:** IM Anuar Ramdhan, W Zulmi, AN Hidayah, MJM Kamel, MSM Fadhil, M Anwar Hau

**Affiliations:** Department of Orthopaedics, Hospital Universiti Sains Malaysia, Kubang Kerian, Malaysia; Department of Orthopaedics, Hospital Universiti Sains Malaysia, Kubang Kerian, Malaysia; Department of Orthopaedics, Hospital Raja Perempuan Zainab II, Kota Bharu, Malaysia; Department of Orthopaedics, Hospital Raja Perempuan Zainab II, Kota Bharu, Malaysia; Department of Orthopaedics, Hospital Raja Perempuan Zainab II, Kota Bharu, Malaysia; Department of Orthopaedics, Hospital Raja Perempuan Zainab II, Kota Bharu, Malaysia

## Abstract

*Background:* Coaptive film (i.e., Steri-Strips™) is an adhesive tape used to replace sutures in wound closure. The use of coaptive film for wound closure after long bone fracture fixation has not been well documented in the literature. *Methods:* The aim of this prospective, randomized controlled trial comparing coaptive film with sutures for wound closure after long bone fracture fixation was skin closure time, incidence of wound complications and scar width at 12 week follow-up. Forty-five patients underwent femur fracture fixation (22 patients’ wound closed with sutures, 23 with coaptive film). *Results:* The mean time for skin closure using coaptive film was 171.13 seconds compared to 437.27 seconds using suture. The mean wound lengths in the coaptive film group and suture group were 187.65 mm and 196.73 mm, respectively. One patient in each group had wound complications. *Conclusion:* Coaptive film is a time-saving procedure for skin closure following long bone fracture fixation. There is no difference in the incidence of wound complications and scar width between these two methods of skin closure.

## Introduction

Coaptive film is an adhesive tape used to replace sutures in wound closure. Van De Gevel et al demonstrated that the Steri-Strip™ brand (3M, St. Paul MN, USA) of coaptive film is a reasonable and safe alternative to sutures without incidence of wound infection, dehiscence or skin irritation^1^. Kerrigan and Homa found that Steri-Strips™ permits faster wound closure than suture^2^, and Lazar et al reported that Steri-StripTM decreases erythema^3^. These above mentioned studies were performed by cardiothoracic surgeons^1^-^3^ or plastic surgeons^2^.

Rebello and colleagues conducted a randomized controlled trial comparing coaptive film (Steri-Strip™) and sutures in children with cerebral palsy undergoing soft tissue releases^4^, and concluded that Steri-Strips™ takes less time to close the skin than sutures; they reported no wound complications and the resulting scars produced similar cosmetic results. These findings were further supported by Grottkau et al who used Steri-Strips™ compare to sutures in children undergoing posterior spinal fusion with instrumentation^5^. They concluded that Steri-Strips™ are time-savers for skin closure following paediatric spine surgery, and produced comparable cosmetic results with no increase in complication rates. We found no reports comparing coaptive film (Steri-StripsTM) to sutures for skin closure in routine orthopaedic trauma surgery.

The difference in skin thickness between the limbs and trunk^6^ can affect wound dehiscence and infection in orthopaedic surgery. The aim of the present study was to compare the use of Steri-Strips™ standard suture technique for wound with closure after long bone fracture fixation, specifically comparing the time taken to close a surgical wound, incidence of wound complications, and scar formation.

## Materials and Methods

*Patients* The study sample consisted of 45 patients, aged 13-33 years old who underwent femoral shaft fracture fixation at a major hospital in Kelantan state Malaysia, from December 2010 to November 2011. Inclusion criterias were closed fracture of the femur that required plating or nailing (open reduction via lateral subvastus approach) requiring primary closure of an operative thigh wound. Exclusion criterias were chronic disease (e.g., diabetes mellitus, chronic renal failure) or prolonged treatment with corticosteroid / chemotherapy drugs for any reason, skin hypersensitivity to coaptive film, open fracture, or history of osteomyelitis in the area of the surgical site. Patients were randomly assigned to either coaptive film technique or suture technique for wound closure after the femoral shaft fracture fixation. The surgeon was informed of closure type at the start of surgery.

*Operative techniques* All patients underwent femoral shaft fracture fixation, either plating or nailing, and the operative wounds were closed in layers. The deep fascial layer was closed with continuous running suture using an absorbable braided synthetic suture (Vicryl 1) and subcutaneous layer was closed with a continuous running suture using absorbable braided synthetic suture (Vicryl 0) to relieve tension and oppose the wound edges.

In the coaptive film group, the skin was closed with Steri- Strips™ (3M, St. Paul, MN USA) whereas in the suture group the skin was closed using interrupted non-absorbable monofilament synthetic suture (Dafilon 3/0). All wounds were dressed and covered with a compressive elastic bandage until wound inspection. The length of time taken to close the skin of the operative wound was recorded in the operation theatre.

*Follow-up* Patients were reviewed and the wound examined at day 3 post-surgery in the ward and subsequently at the end of the 2nd, 6th and 12th-week post-surgery in the orthopaedic clinic as an outpatient. At the 2nd-week visit, wounds were inspected for any wound-related complications (such as inflammation, infection, necrosis or dehiscence). Steri- Strips™ or sutures were removed at this visit. Sutures were removed using a suture cutter or scissors. At the 6th-week visit, wounds were inspected again for any delayed wound related complications. At the 12th-week visit, we evaluated scar formation. Scars were objectively evaluated by measuring the scar width with a Vernier caliper.

*Statistical analysis* We used SPSS software, Version 18 for Window for data analysis. The Fisher’s exact test was used to determine the association of sex, Tscherne classification (for soft tissue injury) and type of surgery with wound closure methods, and also to analyse statistical difference in incidence of wound complication between the two groups. The chi-square test was used to analyze the association of smoking with both groups. The independent t-test was used to determine the association of age in both groups as well as to analyse differences (normal distribution) in wound length, skin closure time and scar width between the two groups. The Mann-Whitney U test was used to analyze the injury-surgery interval in both groups as these data were not normally distributed.

## Results

Of the 45 enrolled patients, we used sutures for 22 patients in the suture group and 23 in the Steri-Strip™ group (Table I). In general, the demographics data shows no significance difference between the two groups for age, sex, smoking status, soft tissue injury following femoral fractures (Tscherne classification) and type of surgery, except for the injury to surgery interval which was significantly different (p=0.022). The Steri-Strip™ group had unintentionally longer intervals from day of injury to the day of surgery with a median of 8 days compared to the suture group with a median interval of 3.5 days.

On the average 2 pack of sutures (suture group) and 3 pack of Steri-Strips™ (Steri-Strip™ group) were used to close the surgical wound . The mean difference in wound length was not statistically significant (p= 0.361) (Table II). Time to complete skin closure time was using Steri-Strips™ faster than sutures (p <0.001).

The incidence of wound complication was noted for each group (Table III), although the difference was not statistically significant. Furthermore, the scar produced by Steri-Strip™ was narrower than that produced by sutures as of the 12th-week follow-up visit (Table II). The difference in scar width was not statistically significant (p= 0.211).

## Discussion

*Skin closure time* This study showed that wound closure using coaptive film (Steri-Strip™) is faster compared to closure using suture technique. By reducing the skin closure time and thus the total operative time, related operative costs may be reduced and the capacity for the number of surgeries performed may also increase. The net effect may benefit the patient as well as our healthcare management and government.

Furthermore, the rapid closure of the skin may also reduce the surgeon’s burden especially after a long surgery, for example a multi-fracture case. With no risk of needle-prick injury, the ease of use of the Steri-Strip™ may reduces stress levels of surgeons and assistants related to wound closure. The shorter skin closure time may also shorten the length of anaesthetic time (for general anaesthesia), and may thereby prevent complications due to lengthy anaesthesia and intubation.

*Wound complication* A majority of study participants had little or no mild soft tissue injury (Tscherne Grade 1). In this study, the Steri- Strip™ group unintentionally had a significantly longer injury to surgery interval time than the suture group. Despite this longer interval, the difference in incidence of wound complication between the two groups was not statistically significant. There was one case of wound complication in each group, and both healed well after local wound care. Therefore, the use of Steri-Strips™ as the wound closure device in post-traumatic long bone surgery is safe and does not entail any additional risk for wound complication.

*car Evaluation* To date, we found no studies describing scar width when using Steri-Strips™. Quinn et al. reported that evaluation of wounds at 3-month post-wound closure provides a good measure of long term cosmetic outcome^7^. Therefore, we assessed the surgical scar for each group at the 12th-week follow-up visit. In the present study, the scar produced by Steri-Strips™ was narrower than seen in the suture group, possibly because Steri-Strip™ causes less skin inflammation than sutures which require multiple needle punctures, strangulation of the wound edges and bridging the surgical wound with a tight knot. It is well known that increased inflammation leads to more scar formation^8^. However, when compared the difference in the scar width was found to be not statistically significant.

**Fig. 1:**
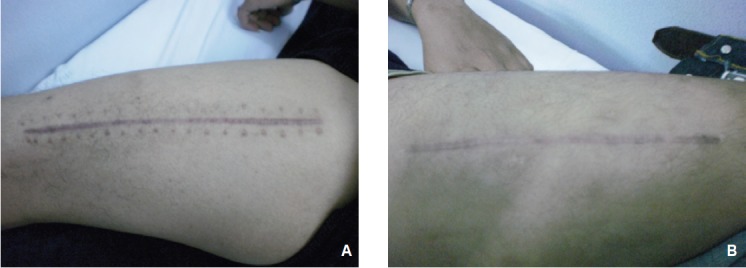
: Photograph, 3 months postoperatively, showing presence of suture and crosshatch marks (A, suture group) and linear scar (B, Steri-Strip™ group).

The presence of suture marks as well as cross-hatching in the suture group, makes the scar cosmetically unpresentable compared to the Steri-StripTM group in which the scar was a simple line (Fig.1). Aesthetics of the scar are important for patients who wear shorts for swimming or other athletics or others who are concerned about the appearance of the scar. The narrower scar width produced by Steri-Strips™ is also more cosmetically pleasing, a further advantages of Steri- Strip™ use as the wound closure device.

*Cost* The average number of packages of Steri-Strips™ and the Dafilon 3/0 sutures used in this study was 3 packs and 2 packs, respectively. Although, the cost for the Steri-Strip™ used was RM 4.00 per pack and the Dafilon 3/0 suture was RM 2.75 per pack, note that material cost mentioned is not the only cost consideration. Steri-Strips™ application does not require specific instruments for removal whereas sutures require scissors or blades for removal. Furthermore, the significant reduction in skin closure time by the Steri-Strip™ reduces the operating theatre time, and may thus decreases surgery cost including materials (e.g., operating monitor, diathermy, suction machines, theatre electricity and medications) and labor (e.g., surgeon, anaesthetist and other surgical staff). If there is time for more cases, Steri-Strip™ used as the wound closure device, may result in overall costsavings. These ‘indirect savings’ due to Steri-Strip™ use as the wound closure device may be realized in all long bone fixation surgeries.

**Table I:**
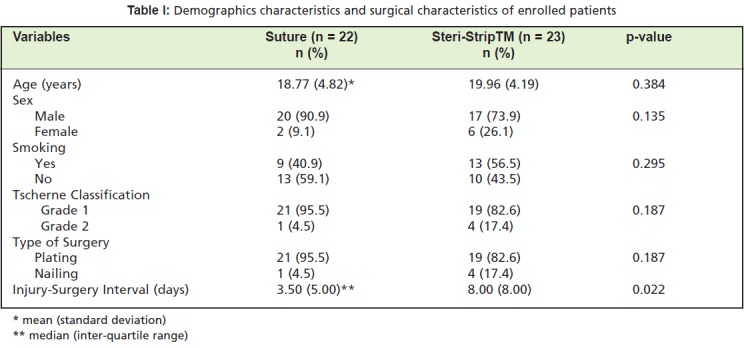
: Demographics characteristics and surgical characteristics of enrolled patients

**Table II:**
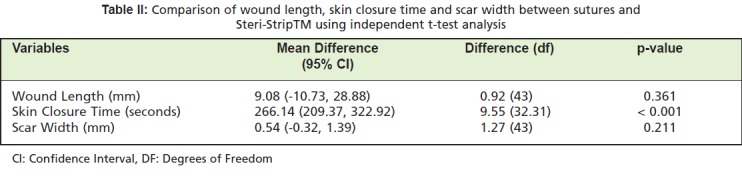
: Comparison of wound length, skin closure time and scar width between sutures and Steri-Strip™ using independent t-test analysis

**Table III:**
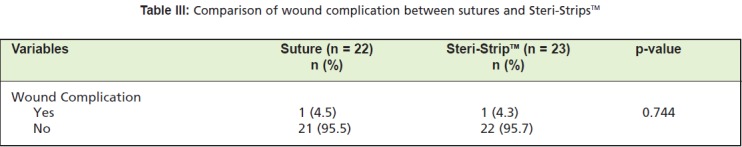
: Comparison of wound complication between sutures and Steri-Strips™

## Conclusion

The use of Steri-StripsTM for wound closure after long bone fracture fixation is a time-saving method that can be safely used without additional risk of postoperative wound complications. Use of Steri-Strips™ resulted in scar width similar to that created by the traditional suture,but provided a better cosmetic outcome with no suture marks or crosshatch marks. Further studies are required to analyse the cost effectiveness of Steri-Strips™ in details and to assess the outcome of the Steri-Strips™ for other fractures fixation sites, especially around mobile joints.
